# Sarcopenia as a prognostic indicator of liver cirrhosis

**DOI:** 10.1002/jcsm.12869

**Published:** 2021-11-23

**Authors:** Sung‐Eun Kim, Dong Joon Kim

**Affiliations:** ^1^ Department of Internal Medicine Hallym University College of Medicine Chuncheon Republic of Korea; ^2^ Institute for Liver and Digestive Diseases Hallym University Chuncheon Republic of Korea

**Keywords:** Sarcopenia, Prognosis, Liver cirrhosis

*Dies annorum nostrum sunt septuaginta anni*

*aut in valentibus octaginta anni*.

Seventy is the sum of our years, or eighty, if we are strong.

‐ Psalm 90,10 ‐

Liver cirrhosis (LC) is widely prevalent and is associated with high mortality, accounting for 3.5% of all deaths worldwide.^1^ Traditionally, LC is divided into compensated cirrhosis (without any symptoms) and decompensated cirrhosis (with the development of complications such as jaundice, ascites, variceal bleeding, or hepatic encephalopathy). In contrast to deaths during the compensated stage, which are largely due to cardiovascular disease, malignancy, and renal disease, deaths during the decompensated stage result from hepatic and extrahepatic organ failure.^1^

The clinical course of LC is not linear but punctuated with episode(s) of acute deterioration (called AD, acute decompensation). To define dynamic syndrome, which is characterized by systemic inflammation, very high short‐term mortality, and rapid reversibility, the term acute‐on‐chronic liver failure (ACLF) has been introduced recently.^2^, ^3^ Patients with ACLF were totally distinct in clinical trajectory from those without ACLF, not only based on the presence of organ failure(s) but also based on mortality rate (33.9% vs. 4.7% in patients without ACLF).^3^ Because ACLF is a distinct clinical entity, in addition to well‐known liver‐specific scores [such as Child–Pugh score and Model for End‐Stage Liver Disease (MELD) score], different prognostic scores (such as the CLIF‐C ACLF score of the EASL, the European Association for the Study of the Liver, the AARC score of the APASL, and the Asian Pacific Association for the Study of the Liver) have been developed.^2^, ^3^

Muscle power has long been recognized as an indicator of life expectancy and was glimpsed even in the Old Testament. Sarcopenia is an important syndrome in aging patients. Patients with chronic disease showed higher prevalence rates of sarcopenia: 29.7% in cardiovascular disease, 24% in chronic obstructive pulmonary disease, and 48.1% in LC.^4^, ^5^ In older adults with cirrhosis, a combination of primary (aging‐related) and secondary (cirrhosis‐related) sarcopenia occurs simultaneously and has been referred to as compound sarcopenia.^6^

Recently, sarcopenia has been defined by the European Working Group on Sarcopenia (EWGSOP) combining low muscle strength, low muscle quantity/quality, and low physical performance.^4^, ^6^ There has been a major change from the original operational definition, as low muscle strength was added as a prerequisite to definitions based only on the detection of low muscle mass. In addition, low physical performance is considered a predictor for poor outcomes; thus, such measures are useful to classify the severity of sarcopenia.^4^

In patients with LC, factors contributing to sarcopenia in an interrelated manner could be categorized as (1) malnutrition (including inadequate intake and uptake; a state of ‘accelerated starvation’), (2) cirrhosis‐related (including synthetic dysfunction; hyperammonaemia, which is myotoxic; anabolic resistance), (3) other systems‐related (including systemic inflammation; gut dysbiosis and altered gut permeability; metabolic dysregulation), (4) physical inactivity, and (5) environmental/organizational factors.^6^

Unlike the geriatric literature, computed tomography (CT) imaging is currently the gold standard for the assessment of muscle mass in cirrhosis.^6^, ^7^ When abdominal CT imaging is performed for clinical reasons, muscle mass measurement can be obtained using quantitative morphomics software as the skeletal muscle mass (SMI) at the third lumbar vertebra (L3).^6^ Optimal cut‐off values of L3‐SMI derived from cirrhotic patients were established as 50 cm^2^/m^2^ for men and 39 cm^2^/m^2^ for women by the EASL.^8^ Considering that the mean muscle mass of Asians is approximately 15% lower than that of individuals from Western populations, it is necessary to propose other cut‐off values for Asian populations. The Japan Society of Hepatology proposed the cut‐off values of L3‐SMI as 42 cm^2^/m^2^ for men and 38 cm^2^/m^2^ for women.^9^ Zeng *et al*.^10^ suggested different cut‐off values for the L3‐SMI in Chinese adults, defining sarcopenia as 44.77 cm^2^/m^2^ for men and 32.5 cm^2^/m^2^ for women.

Similar to the general population, there are strong sex‐based differences in the prevalence of sarcopenia, with 21% of women and 54% of men with cirrhosis awaiting liver transplantation (LT), and the degree of muscle loss correlates with the severity of liver disease in men but not in women.^6^ Meanwhile, the aetiology of liver disease has been associated with differences in the prevalence of sarcopenia.^6^ For example, alcoholic liver disease has been associated with a high prevalence of sarcopenia, affecting 80% of patients with decompensated cirrhosis, although sarcopenia was reported in approximately 60% of patients with cirrhosis from non‐alcoholic steatohepatitis, chronic hepatitis C virus infection, and autoimmune hepatitis.^6^, ^11^, ^12^ In Zeng's study,^10^ the aetiologies of 480 patients with cirrhosis were hepatitis B in 50.34% of males/32.07% of females, alcohol in 20.27%/0.54%, autoimmune in 0.34%/20.65%, and others 16.55%/38.59%.

Sarcopenia has been shown to be a robust predictor of a wide spectrum of outcomes, such as hepatic decompensation, quality of life, risk for infection, and mortality, in patients with LC, patients with post‐LT,^4^, ^13^ and patients with hepatocellular carcinoma.^14^ Interestingly, the impact of sarcopenia on survival has mainly been shown in cirrhotic patients with Child–Pugh class A or B and patients with low MELD scores. In a Canadian study of 669 cirrhotic patients who were evaluated for LT, Montano‐Loza *et al*. reported that the modification of MELD to include sarcopenia (MELD‐sarcopenia) is associated with an improvement in the prediction of mortality in patients with cirrhosis, and the observed benefit of modifying MELD to include sarcopenia was greatest in patients with low MELD scores, who are traditionally deemed to have a low risk of death.^15^ Similarly, in a Korean study, although cirrhotic patients with sarcopenia had higher MELD scores, sarcopenia was associated with mortality in compensated and early decompensated cirrhosis.^16^ Kang *et al*. showed that the impact of sarcopenia was stronger in patients with low MELD scores (MELD score < 15), Child–Pugh class A/B, and hepatic venous pressure gradient (HVPG) < 20 mmHg. In contrast, there was no association with sarcopenia and mortality in patients with high MELD scores, Child–Pugh class C, and HVPG ≥ 20 mmHg.^16^ These findings that sarcopenia has little additive benefit on mortality prediction for patients who are too ill are suggested by previous studies. For example, in a prospective Italian study of 1053 cirrhotic patients to determine whether malnutrition is a risk factor for mortality, Merli *et al*. found that cumulative survival was lower in patients with a reduction in muscle mass in Child–Pugh classes A and B but not in class C.^17^ In the most severe patients, the influence of sarcopenia on survival was not found, probably because their survival is threatened in the short term by other factors. Zeng *et al*.^10^ demonstrated a different finding: Higher MELD or Child–Pugh class C patients with sarcopenia had poorer overall survival than those without sarcopenia (both *P* < 0.001). They reported that sarcopenia was correlated with Child–Pugh class, MELD score, and mortality. However, cirrhotic patients with Child–Pugh class C or higher MELD scores are prone to progress to ACLF. In a recent study of US veterans, Shah *et al*. demonstrated that frailty increases the likelihood of ACLF hospitalization among patients with cirrhosis, but it does not impact short‐term ACLF mortality.^18^ These findings may be because once ACLF has been triggered, the most critical factor might be organ dysfunction, which drives mortality, as opposed to risk factors leading to inciting ACLF events, such as sarcopenia and frailty. Therefore, there might be a possibility of errors in determining the independent effect of sarcopenia on mortality in patients with Child–Pugh class C or high MELD, who are prone to develop ACLF if they are not analysed separately.

Sarcopenia is not only a common but also a clinically significant condition that occurs in patients with LC.^19^ Future studies of sarcopenia in patients with chronic liver disease should include the following: (1) There are insufficient data for cut‐offs of sarcopenia in different ethnicities, ages, sexes, and so forth. (2) The significance of sarcopenia should be explored in terms of aetiology (e.g. alcoholic vs. non‐alcoholic). More importantly, patients with LC should be stratified, not only by compensated vs. decompensated but also by the presence vs. absence of AD and/or ACLF. In addition, the effects of contextual factors, such as ambulatory vs. in‐hospital patients and pre‐LT waiting vs. post‐LT waiting, on the prognostic value of sarcopenia should be investigated. (3) Considering that sarcopenia is a progressive and dynamic state, longitudinal and serial measurements are required. It is recommended that the reassessment of sarcopenia should occur at least annually for patients with well‐compensated disease but as frequently as every 8 to 12 weeks in those with decompensated cirrhosis.^6^ (4) Both frailty and sarcopenia might be more relevant as complementary endpoints in research studies.^6^ (5) It is also necessary to determine whether an improvement in sarcopenia independently impacts the course of LC or whether an improvement is secondary to an improvement in liver function.^19^

## Conflict of interest

The authors declare that no conflict of interest relevant to this article exists.
